# Supplementary material for: TGF-β/Smad Signalling Activation by HTRA1 Regulates the Function of Human Lens Epithelial Cells and Its Mechanism in Posterior Subcapsular Congenital Cataract
Source: Int J Mol Sci. 2022 Nov 20;23(22):14431. doi: 10.3390/ijms232214431 (PMC9692351; doi:10.3390/ijms232214431)
Supplement: Supplementary file 1 [file ijms-23-14431-s001.zip › ijms-1972724-supplementary.pdf]

Table S1. Primers for RT-qPCR assay.

Target	F/R	Primer Sequence (5'→3′)	Amplicon Size (BP)
TGFB1	Forward	CTAATGGTGGAAACCCACAACG	209
	Reverse	TATCGCCAGGAATTGTTGCTG	
TGFB2	Forward	GAGAGGAGCGACGAAGAGTA	687
	Reverse	CTCAAGTCTGTAGGAGGGCA	
TGFBR1	Forward	CACAGAGTGGGAACAAAAAGGT	143
	Reverse	CCAATGGAACATCGTCGAGCA	
PAI 1	Forward	ACCGCAACGTGGTTTTCTCA	109
	Reverse	TTGAATCCCATAGCTGCTTGAAT	
CTGF	Forward	ACCGACTGGAAGACACGTTTG	195
	Reverse	CCAGGTCAGCTTCGCAAGG	
HTRA1	Forward	TCCCAACAGTTTGCGCCATAA	119
	Reverse	CCGGCACCTCTCGTTTAGAAA	
XBP-1s	Forward	CTGAGTCCGAATCAGGTGCAG	59
	Reverse	ATCCATGGGGAGATGTTCTGG	
ATF6	Forward	TCGGTCAGTGGACTCTTATT	153
	Reverse	CCAGTGACAGGCTTATCTTC	
IRE1	Forward	GGACAGGCTCAATCAAATGG	255
	Reverse	CGGTCAGGAGGTCAATAACA	
BIP	Forward	CATCACGCCGTCCTATGTCG	104
	Reverse	CGTCAAAGACCGTGTTCTCG	
FN1	Forward	CGGTGGCTGTCAGTCAAAG	130
	Reverse	AAACCTCGGCTTCCTCCATAA	
VIM	Forward	GACGCCATCAACACCGAGTT	238
	Reverse	CTTTGTCGTTGGTTAGCTGGT	
α-SMA	Forward	TGCCAACAACGTCATGTCG	79
	Reverse	CAGCGCGGTGATCTCTTTCT	
GAPDH	Forward	GGAGCGAGATCCCTCCAAAAT	197
	Reverse	GGCTGTTGTCATACTTCTCATGG	

Table S2. Antibody list

Protein	Brand	Catalog number	Dilution
HTRA1	Abcam, UK	Ab274322	1:1000 for WB<br>1:100 for IF
HRP-conjugated β-actin	Proteintech, USA	HRP-60008	1:5000 for WB
Bcl2	Proteintech, USA	26593-1-AP	1:1000
BAX	Proteintech, USA	50599-2-Ig	1:1000
Cleaved-caspase-3	CST, USA	9661	1:1000
SMAD 2/3	Abcam, UK	Ab202445	1:1000
p-SMAD 2/3	Abcam, UK	Ab254407	1:1000
TGF-β1	Proteintech, USA	21898-1-AP	1:1000 for WB<br>1:200 for IF
TGF-β2	Proteintech, USA	19999-1-AP	1:1000 for WB<br>1:200 for IF
FN1	Proteintech, USA	15613-1-AP	1:2000
α-SMA	CST, USA	19245	1:1000
PAI-1	Proteintech, USA	13801-1-AP	1:1000
CTGF	Proteintech, USA	25474-1-AP	1:1000
TGF-βR1	Abcam, UK	Ab235578	1:1000
HRP-labelled Goat Anti-Rabbit IgG(H+L)	Beyotime	A0208	1:1000
HRP-labelled Goat Anti Mouse IgG(H+L)	Beyotime	A0216	1:1000
Anti-rabbit, Secondary Antibody, Alexa Fluor 488	Abcam, UK	Ab150077	1:1000
